# Data on color and chemical composition of dried scallop (*Mizuhopecten yessoensis*) produced in different areas of Hokkaido, Japan

**DOI:** 10.1016/j.dib.2017.11.077

**Published:** 2017-11-27

**Authors:** Hisaki Enda, Yoshimasa Sagane, Yozo Nakazawa, Hiroaki Sato, Masao Yamazaki

**Affiliations:** a Kohken Food and Flavor Co. Ltd., 2-3-19 Takashima, Nishi-ku, Yokohama 220-0011, Japan; b Department of Food and Cosmetic Science, Faculty of Bioindustry, Tokyo University of Agriculture, 196 Yasaka, Abashiri, Hokkaido 099-2493, Japan

**Keywords:** Quality of products, Dried scallop, Seasoning ingredient, Browning

## Abstract

Dried scallop is used in Chinese, Japanese, and French cuisines for its unique flavor and taste. The quality of dried scallop is rated according to its clear, shiny brown color, developed by the Maillard reaction between sugars and amino acids. This article reports the colors, represented by L* and a* values, and chemical composition (water, salinity, Brix, proteins, and amino acids) of dried scallop products. The dried scallops were produced in Tokoro, Sarufutsu, and Saroma in Hokkaido, Japan. The color of the dried scallops had values of 45.7–52.0 for L* and 2.31–5.08 for a*. The salinity of the products was 15.1–17.7%. The amino acid contents were 1350.8–1668.6 mg/100 g. The data collected here are provided in table format. The data can serve as a reference for commercial dried scallop products to determine product quality.

**Specification Table**

**Table:** 

Subject area	Agricultural science
More specific subject area	Food chemistry
Type of data	Table
How data were acquired	Colors of dried scallop products were measured using a colorimeter, and chemical compositions were measured using salt meter, Brix meter, amino acid analyzer, and methods published by the Association of Official Analytical Chemists.
Data format	Raw, analyzed
Experimental factors	Dried scallop products were purchased at the market
Experimental features	Colors as represented by L* and a* values, and contents of water, salt, Brix, protein, and amino acids
Data source location	Tokoro, Sarufutsu, and Saroma, Hokkaido, Japan
Data accessibility	Data are presented in this article

.

**Value of the data**

• The data on the color of dried scallop products can serve as reference for commercial dried scallop products to determine product quality.
• The data will be useful for nutrient assessment of dried scallop products, based on their chemical components.
• The data presented would be useful to predict the effects of culture sites of scallop materials and drying processes in each area on the chemical and color characteristics of the products.

## 1 Data

Colors of the dried scallop products were measured using a colorimeter and represented as L* and a* values (Table 1). Salt and water contents, Brix values, protein contents, and total amino acid contents in the products are also provided in Table 1. The free amino acid composition is listed in Table 2.

**Table 1:** Colors and chemical compositions of dried scallop products.

Production		Color (*n*=20)		Salinity	Water (*n=*6)	Brix (%)	Protein (g/100 g)	Amino acid (mg/100 g)
Origin	Year	L*	a*	(%)	(%)			
Tokoro	2014	52.0±1.16	2.3±0.64	2.9	17.7±1.4	10.1	62.2	1668.6
Tokoro	2015	47.1±1.16	2.5±0.66	3.1	16.6±1.8	9.1	45.5	1381.1
Sarufutsu	2014	48.5±2.29	5.1±0.66	3.2	17.1±0.3	10.2	65.2	1432.4
Sarufutsu	2015	43.4±0.92	3.5±1.26	3.3	16.7±0.3	9.2	46.8	1350.8
Saroma	2015	45.7±2.14	4.1±0.44	3.0	15.1±1.0	9.7	47.5	1541.5

**Table 2:** Free amino acid composition of dried scallops. Values are presented as the % of total amino acids.

Amino acids	Tokoro		Sarufutsu		Saroma
	*2014*	*2015*	*2014*	*2015*	*2015*
Taurine	27.0	29.1	34.0	33.2	33.3
Aspartic acid	n.d.^a^	n.d.	n.d.	n.d.	n.d.
Threonine	0.6	0.7	0.3	1.0	0.7
Serine	0.2	0.1	0.1	0.1	0.2
Asparagine	n.d.	n.d.	n.d.	n.d.	n.d.
Glutamic acid	2.4	2.7	2.2	3.2	2.5
Glutamine	0.2	0.3	n.d.	0.3	0.2
Proline	n.d.	n.d.	n.d.	n.d.	n.d.
Glycine	44.6	43.6	40.0	39.3	41.6
Alanine	4.7	5.8	3.0	4.5	3.5
Citrulline	n.d.	n.d.	n.d.	n.d.	n.d.
Valine	0.4	0.5	0.3	0.5	0.4
Cysteine	n.d.	n.d.	n.d.	n.d.	n.d.
Methionine	0.3	0.5	0.3	0.8	0.5
Isoleucine	0.2	0.2	0.2	0.2	0.2
Leucine	0.3	0.2	0.2	0.3	0.3
Tyrosine	n.d.	n.d.	n.d.	n.d.	n.d.
Phenylalanine	n.d.	0.1	n.d.	0.2	0.1
Ornithine	n.d.	n.d.	n.d.	n.d.	n.d.
Lysine	0.4	0.3	0.4	0.3	0.2
Histidine	0.1	0.2	0.0	0.2	0.1
Arginine	18.6	15.7	19.0	15.9	16.4
Total	100.0	100.0	100.0	100.0	100.0

a n.d.: not detected.

## 2 Experimental design, materials, and methods

### 2.1 Design

Dried scallop products are eaten as is without cooking or are used as seasoning in Japanese, Chinese, and French cuisines. The scallop adductor turns brown because of the Maillard reaction between sugars and amino acids in the material adductor muscle during the drying process, and the clear, shiny brown color is an indicator of a high-quality product [1]. In this study, therefore, the color and salt, water, sugar, and amino acid contents related to the quality of dried scallop products were measured. For the color measurement, the *L value that represents brightness and *a value that represents red/green were employed to assess lightness and degree of browning of the products, respectively.

### 2.2 Materials

Dried scallop products produced in Tokoro, Sarufutsu, and Saroma (Hokkaido, Japan) in 2014 and 2015 were purchased at a local market.

### 2.3 Color analysis

The colors of dried scallop products were measured using a colorimeter (CR-400; Konica Minolta, Tokyo, Japan). The colors of the products (two parts for each of 10 products) were measured to determine L* and a* values.

### 2.4 Water measurement

To determine the water content of the dried scallop products (6 products from each sample), the scallop was dried at 105 °C for 16 h. The difference in weights before and after drying was defined as water content and is expressed as a % of the original weight.

### 2.5 Salinity, Brix, and protein content measurement

The scallops (60 g) were boiled in twice the volume of water at 5 °C for 19 h. The salinity of the extract was determined using a salt meter (SALTMETER ESSALTMETER ES-421; Atago Co. Ltd., Tokyo, Japan). Brix in the extract was analyzed using a Brix meter (PR-101a; Atago Co. Ltd.). The amount of protein in the extract was determined by the micro-Kjeldahl method using a Kjeltec 2200 system (Foss, Hillerød, Denmark). The protein amount was measured using a conversion factor of 6.25.

### 2.6 Amino acid analysis

The extract was mixed with same volume of 5% trichloroacetic acid (TCA) to precipitate the proteins contained in the sample solution. After centrifugation (10,000 rpm, 10 min) the supernatant was mixed with same volume of n-hexane, and the mixture was allowed to stand for a few minutes, after which it was filtered through a cellulose acetate membrane (0.2 µm). The amino acid concentration in the filtrate was determined using a LaChrom Elite amino acid analysis system (Hitachi High-Technologies Corp., Japan) by a post-column ninhydrin method, as previously reported [2]. The temperature for the ninhydrin reaction was set to 130 °C (reagent flow rate: 0.25 ml/min). The reacted amino acids were detected by recording the absorbance at 570 nm.
